# Deleterious Effects of Heat Stress on the Tomato, Its Innate Responses, and Potential Preventive Strategies in the Realm of Emerging Technologies

**DOI:** 10.3390/metabo14050283

**Published:** 2024-05-15

**Authors:** Qaisar Khan, Yixi Wang, Gengshou Xia, Hui Yang, Zhengrong Luo, Yan Zhang

**Affiliations:** Department of Landscape and Horticulture‚ Ecology College‚ Lishui University‚ Lishui 323000‚ China; qaisar.khan@yahoo.com (Q.K.); yxwangls@163.com (Y.W.); lsxyxgs@163.com (G.X.); lsxyyh@126.com (H.Y.); zrluo@126.com (Z.L.)

**Keywords:** heat stress, reactive oxygen species, heat shock proteins, stress signaling, genome editing, omics, heat tolerance pyramiding, genetic resources

## Abstract

The tomato is a fruit vegetable rich in nutritional and medicinal value grown in greenhouses and fields worldwide. It is severely sensitive to heat stress, which frequently occurs with rising global warming. Predictions indicate a 0.2 °C increase in average surface temperatures per decade for the next three decades, which underlines the threat of austere heat stress in the future. Previous studies have reported that heat stress adversely affects tomato growth, limits nutrient availability, hammers photosynthesis, disrupts reproduction, denatures proteins, upsets signaling pathways, and damages cell membranes. The overproduction of reactive oxygen species in response to heat stress is toxic to tomato plants. The negative consequences of heat stress on the tomato have been the focus of much investigation, resulting in the emergence of several therapeutic interventions. However, a considerable distance remains to be covered to develop tomato varieties that are tolerant to current heat stress and durable in the perspective of increasing global warming. This current review provides a critical analysis of the heat stress consequences on the tomato in the context of global warming, its innate response to heat stress, and the elucidation of domains characterized by a scarcity of knowledge, along with potential avenues for enhancing sustainable tolerance against heat stress through the involvement of diverse advanced technologies. The particular mechanism underlying thermotolerance remains indeterminate and requires further elucidatory investigation. The precise roles and interplay of signaling pathways in response to heat stress remain unresolved. The etiology of tomato plants’ physiological and molecular responses against heat stress remains unexplained. Utilizing modern functional genomics techniques, including transcriptomics, proteomics, and metabolomics, can assist in identifying potential candidate proteins, metabolites, genes, gene networks, and signaling pathways contributing to tomato stress tolerance. Improving tomato tolerance against heat stress urges a comprehensive and combined strategy including modern techniques, the latest apparatuses, speedy breeding, physiology, and molecular markers to regulate their physiological, molecular, and biochemical reactions.

## 1. Introduction

The tomato, scientifically known as *Solanum lycopersicum* in the Solanaceae family, is cultivated in diverse environmental circumstances and geographical regions ranging from tropical to temperate environments. The tomato arrived in Europe during the Renaissance and was scattered to the Mediterranean region [1]. The tomato is a fruit vegetable among the most cultivated crop plants on the earth and is grown in greenhouses and fields worldwide [2]. It is rich in medicinal and nutritional contents, including lycopene, the valuable compound having anti-oxidative and anti-cancer properties, vitamins A and C, β-carotene, iron, phosphorus, flavonoids, ferulic acid, hydroxycinnamic acid, chlorogenic acid, homovanillic acid, folate, and low calories [3,4,5,6]. Around 80% of tomatoes are used as processed food like ketchup, soup, paste, sauces, and juices [7,8]. Globally, China is the biggest producer of tomatoes, followed by India and Turkey (FAO-2021) [9].

Earlier researchers have studied and discussed numerous facets of heat stress on tomatoes, which include plant growth, leaf morphology, photosynthesis, and reproductive performance, including fruit sets, root growth, ROC species, pollen viability, pollen numbers, and inflorescence numbers, focusing on individual aspects. In the context of global warming, this current review provides a thorough critical analysis of heat stress on tomatoes, covering all major aspects, including seed germination, growth, and development and physiological, biochemical, genetic, and molecular reactions. Furthermore, it offers comprehensive information about the available technologies and potential approaches for creating imminent heat-tolerant cultivars. This present review provides complete insight into all significant negative aspects of heat stress on tomatoes, their morphological, physiological, biochemical, and molecular responses, analytical methodologies, and strategies for developing heat-tolerant tomato cultivars.

## 2. Heat Stress

The undesirable influence of non-living dynamics and factors on living organisms in a specified environment is termed abiotic stress [10]. Several abiotic stresses, such as heat, flood, drought, and salt, reduce the production and yield of tomato crops by up to 75%; particularity is subjected to the severity of stresses [11]. Generally, heat stress is defined as an increase in temperature beyond tolerance for an unknown duration, adequate to trigger irretrievable impairment in plant growth and development. In contrast, heat tolerance is defined as a plant’s capability of growth and production to an economic yield level under high temperatures [12,13]. In the context of tomato cultivation, heat stress is commonly classified as moderate heat stress, ranging from 32 °C to 37 °C, and severe heat stress, ranging from 38 °C to 45 °C [14]. Climate changes drastically affect tomato crop production and yield, particularly in Asian countries [15]. It is a common opinion that soaring temperatures will enhance the average temperature of the earth’s surface by 0.2 °C every ten years in the coming thirty years, increasing extreme weather and, consequently, negatively affecting tomato plant growth and development and severely reducing its production and yield [16,17].

## 3. Negative Effects of Heat Stress on Growth and Development

Tomato plants can typically grow and develop reproductive organs, pollen grains, and fruit sets at an optimum temperature between 15 °C and 32 °C; however, temperatures beyond 35 °C badly stress sexual and asexual development [18,19]. Being sessile, tomato plants often face erratic high-temperature conditions, which adversely influence them as temperatures go beyond the optimal ranges. Studies have revealed that high temperatures increase the frequencies of hot and dry days, affecting tomato plant growth, biomass, phenology, agronomic traits, production, and yield [20,21]. High temperature significantly disrupted physiological characteristics such as leaf water content, membrane stability, canopy temperature drop, photosynthesis, stomatal conductance, chlorophyll content, and fluorescence [22,23]. High heat stress negatively influences the metabolic processes involved in growth and development [24]. It produces reactive oxygen species, like hydrogen peroxide (H_2_O_2_), superoxide, hydroxyl radical (OH), and singlet oxygen 1[O2], which adversely disturb cellular homeostasis [25,26,27].

In numerous crops, particularly tomatoes, reproductive growth is highly prone to heat stress compared with vegetative growth [28]. Seeds are a significant part of plants, which carry genetic information to descending generations [29]. However, higher temperatures seriously threaten seed germination, seedling physiology, and phenotypic expression [30,31]. Seed germination tested at a constant range of temperatures from 24 °C to 37 °C for 8 days showed that the rate of seed germination started reducing after 28 °C and entirely ceased at 36 °C. Cotyledon size reduced at a temperature higher than 24 °C but the seedling’s hypocotyl length increased by 1.9 cm (24 °C), 4.1 cm (28.5 °C), and 2.6 cm (31.5 °C), which shows that temperatures higher than 28.5 °C also affect hypocotyl length negatively. In the same study, tomato seedlings aged 12 days (germination: 24 °C) were exposed to 37 °C for 24 h, and a 1 h heat wave (45 °C) damaged the seedling’s recovery ability. Exposure to a 45 °C heat wave for 1 h, 3 h, 6 h, and 12 h showed that the seedlings started drying at 6 h and lost recovery capability at 12 h. The number of lateral roots was reduced, but the growth of the primary root was stopped at 37 °C. Although 45 °C did not affect lateral roots significantly, it halted the growth of the primary root [32,33]. High temperature reduces tomato root growth and nutrient uptake, affecting root–shoot source–sink relationships that affect fruit yield and quality [28,34]. The 30-day-old seedlings of two tomato cultivars (Dafnis and Minichal) were subjected to heat stress of 40 °C for 7 days in a growth chamber, and the results indicated that the effects of high temperature on tomato leaves started to appear on the second day. However, a big difference was noticed on the seventh day. The damage to the leaves of the Dafnis cultivar was over 60%, but Minichal showed resistance [35], which suggests that heat is a serious problem for tomato plants, and the creation of heat-resistant varieties is very important to avoid economic losses.

## 4. Adverse Impacts of Heat Stress on Photosynthetic Parameters

**Figure 1:**
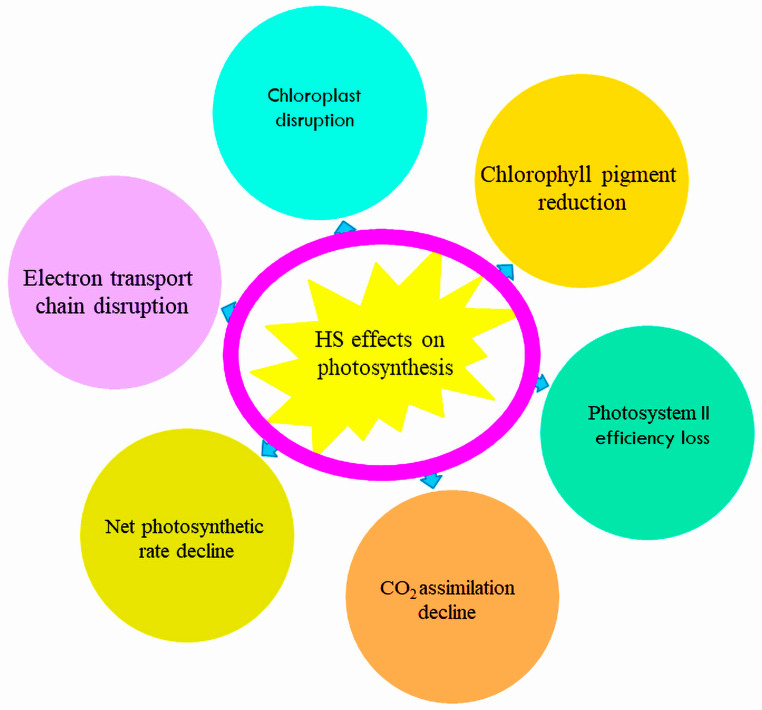
The negative impacts of heat stress on photosynthetic parameters.

Exposure of tomato plants to higher temperatures leads to significant disruption of the chloroplast, which produces adenosine triphosphate (ATP) and phytochemicals. A good performance of the photosynthetic apparatus under high-temperature stress shows the ability of a plant to tolerate and adapt to stressful conditions [36,37]. However, HS negatively affects several important components of photosynthesis (Figure 1). Heat stress inhibits chlorophyll formation; hence, measuring chlorophyll (a, b) concentrations can be a parametric indication for identifying heat-resistant plants. Under directly applied high-temperature stress of 45 °C (severe stress) for 2 h, a heat-resistant tomato cultivar showed a decline in the ratio of chlorophyll (a:b) and an increase in the ratio of chlorophyll to carotenoid in contrast to the control condition of 25/20 °C (day/night). Heat-sensitive cultivars, on the other hand, showed a decrease in the CO_2_ assimilation rate (A), the net photosynthetic rate (Pn), and photosystem II efficiency (Fv/Fm), which represents the highest quantum efficacy of photosystem II (PSII) and is used to assess chloroplasts’ normal or superior functioning under heat stress conditions [38,39,40,41,42].

Photosynthesis in plants is a heat-sensitive physiological process that influences chlorophyll content, CO_2_ integration, D1 and D2 protein turnover, chloroplast components, and heat-responsive protein deactivation [43]. Plant growth, development, production, yield, and future food security are deeply connected with photosynthesis [44,45]. Persistent higher heat stress inhibits photosynthetic activities, which affect the growth and production of plants. Photosystems I and II (PSI, II), chlorophyll, the electron transport chain, and CO_2_ assimilation are among the significant photosynthesis process components, so damage to any of them retard the photosynthetic mechanism [46]. A study by reference [47] revealed that the production of protochlorophyllide (Pchlide), an intermediate in the biosynthetic pathway of chlorophyll, was repressed by 70% at high temperature (42 °C) compared with a control (25 °C), which reduced chlorophyll manufacture to 60% in cucumber seedlings. Similarly, in the same study, the activities of the 5-aminolevulinic acid dehydratase (ALAD) enzyme, which is responsible for converting 5-aminolevulinic acid (ALA) into porphobilinogen (PBG), an intermediate in chlorophyll biosynthesis, and porphobilinogen deaminase (PBGD), which is necessary for converting PBG into urogen, were reduced by 45% and 28% at a higher temperature compared with a control. The PSII electron transport system is highly vulnerable to high temperature because it increases thylakoid membrane fluidity, which knockouts the PSII light-garnering system from the thylakoid membrane and, consequently, destroys PSII integrity [48,49]. High temperature severely disturbs tomato plants’ photosynthetic activities, specifically in susceptible tomato varieties [50].

## 5. Heat Stress Represses Reproductive Performance

**Figure 2:**
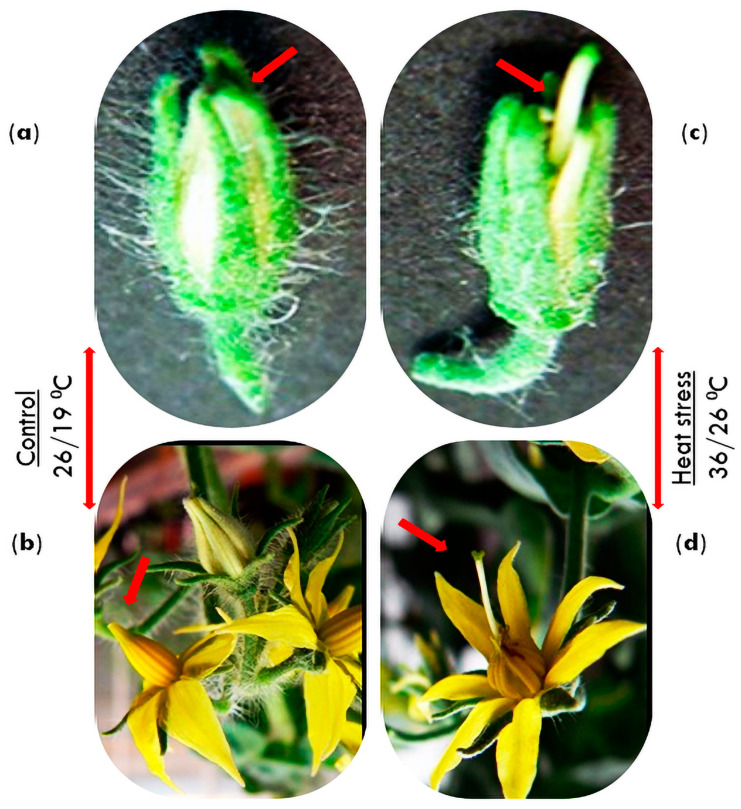
Phenotypic changes in the tomato (cv. Saladette) flowers subjected to heat stress. (**a**,**b**) are young flower buds and flowers at the blooming stage under normal temperatures (26/19 °C). (**c**,**d**) are flower buds and opened flowers under heat stress (36/26 °C).

Cultivated tomatoes are autogamous plants, and high temperatures negatively impact their pollination [51]. Under high heat stress, the tomato style, which is the female reproductive part of the flower gynoecium holding the stigma, extends abnormally and goes out the antheridial cones, minimizing the chances of pollination and, consequently, reducing fruit sets (Figure 2) [52,53,54,55]. Heat stress distorts pollen grain development by reducing the amount of carbohydrates at the early stages of development, reducing the sugar concentration in mature pollens, and resulting in slashed pollen viability [56,57]. The responses of heat-resistant and susceptible genotypes of *Lycopersicon esculentum* Mill. and *L*. *pimpinellifolium* Mill. to heat stress was evaluated by subjecting plants to optimal (27/23 °C, day/night) and high-temperature (35/23 °C) regimes in a greenhouse. The heat tolerance ratings of the genotypes were determined by calculating the percentage of fruit that successfully developed under high and optimal temperatures. The fruit sets varied from 41% to 84% in the temperature-sensitive genotypes and 45% to 91% in the heat-tolerant genotypes at optimal temperatures. The genotypes with great heat sensitivity did not yield any fruit. In contrast, the genotypes that could withstand high temperatures produced fruit set rates ranging from 45% to 65% [58]. Stigma and stylar exsertion negatively affect fruit set forming capabilities because of elevated temperatures [59]. It is essential to create heat-resistant tomato varieties with higher fruit sets as these varieties will benefit tomato crop yield in areas where the growing season’s average temperature is 35 °C or higher.

## 6. Negative Impacts of Heat Stress on Agronomic Traits

Agronomic traits refer to the characteristics of plants that exert influence on their productivity, quality, and ability to cope with biotic and abiotic stressors. The adverse effects of heat stress hinder the overall capacity of tomatoes to reach the desired agronomic performance. Several investigations have been carried out to assess the negative impacts of heat stress on different aspects of tomato leaves, such as fresh mass, the leaf area, the leaf area ratio, the specific leaf area, and plant height and stem diameter under multiple heat stress levels [60,61]. The total area of all leaves on a single plant is referred to as the leaf area (LA) [62]. The specific leaf area (SLA) is a crucial statistic for plant growth modelers as it specifies the amount of fresh leaf area to allocate for each unit of biomass produced; it is calculated by dividing the leaf area by the leaf mass (LA/LM) [63]. Heat stress negatively affects plant leaves in several other ways, including reducing their capacity to retain water and early leaf mortality [64,65]. Heat stress causes glucose reserve shortages because it impedes starch accumulation, which results in a decrease in soluble sugar concentration obtained from the decomposition of starch in fully developed pollen grains [66]. These incidents can potentially decrease tomato pollen fertilization capacity [67]. An increase in diurnal temperature over 25 °C adversely impacted fruit quantity, weight, and seed count per fruit markedly [68].

### Heat Stress and Heat Shock Combined Effects

**Figure 3:**
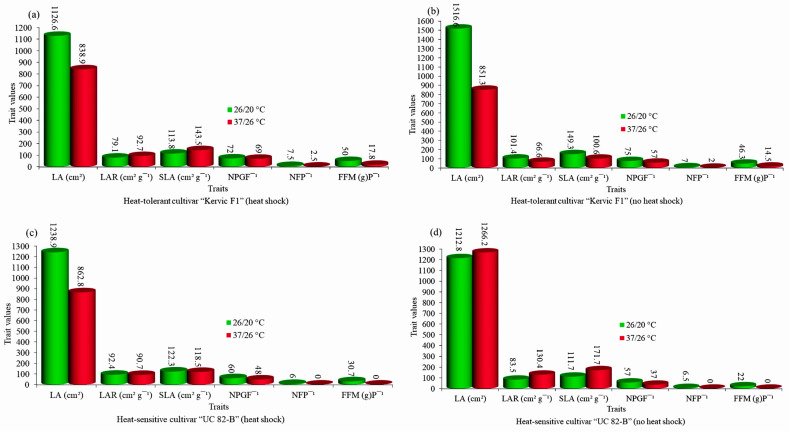
Effects of heat stress and heat shock on the agronomic parameters of resistant (Kervic F1) and sensitive (UC 82-B) tomato cultivars. (**a**) Heat-resistant cultivar (Kervic F1) under heat stress after heat shock stress. (**b**) Heat-resistant cultivar (Kervic F1) under heat stress without heat shock stress. (**c**) Heat-sensitive cultivar (UC 82-B) under heat stress after heat shock stress. (**d**) Heat-sensitive cultivar (UC 82-B) under heat stress without heat shock stress. The X-axis indicates the types of agronomic parameters investigated, and the Y-axis shows values of changes in agronomic parameters under heat stress.

The tomato cultivars Kervic F1 (heat-resistant) and UC 82-B (heat-susceptible) at the age of 35 days after heat shock at 50 °C for 30 s were subjected to a heat stress of 35/27 °C (day/night) compared to control 26/20 °C (day/night) conditions to study agronomic traits including the leaf area (LA), leaf area ratio (LAR), specific leaf area (SLA), number of pollen grains per flower (NPGF), number of fruits per plant (NFP), and fruit fresh mass per plant (FFMP) [69]. Heat stress and heat shock negatively influenced the agronomic traits of tomatoes, particularly the leaf area, pollen grains, fruit sets, and fruit weight (Figure 3). The heat stress repercussions mentioned herein hinder the overall capacity of tomatoes to perform better agronomically. Therefore, it is crucial to extensively examine all physiological and agronomic characteristics to address heat stress issues effectively.

## 7. Over Production of Reactive Oxygen Species (ROS)

**Figure 4:**
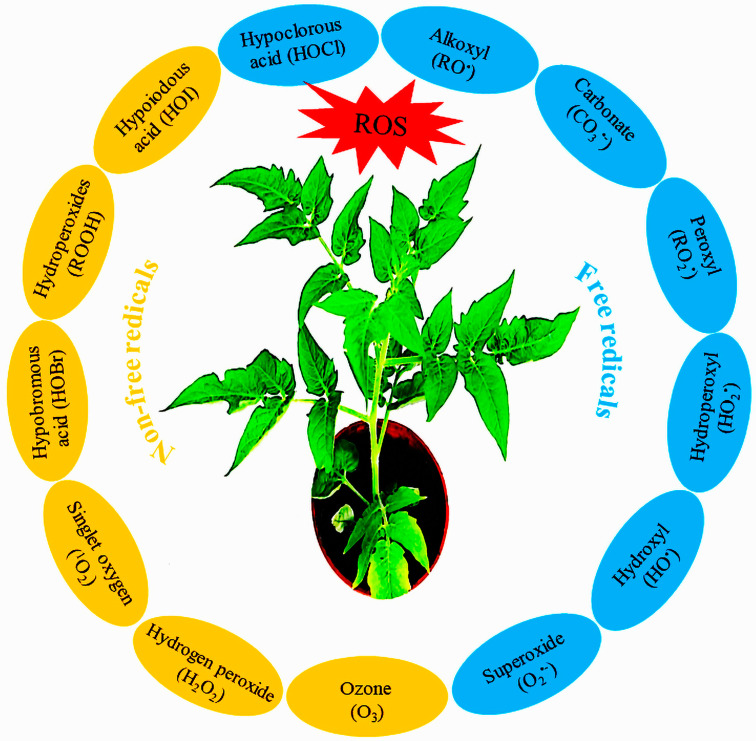
A display showing various types of reactive oxygen species functioning in tomato plants.

An equilibrium among numerous pathways in diverse cell compartments maintains cellular homeostasis under an optimal temperature. The sustainability of homeostasis cannot be guaranteed when temperatures go beyond the optimal level because various pathways have diverse optimum temperatures within the cell, and heat stress upsets this functional balance between different pathways [70]. ROS are over-produced in response to high-temperature stress and other harmful factors that affect several intracellular pathways [71,72]. ROS include free and non-free radicals that contain oxygen and are capable of self-regulating survival with one or more unpaired electrons (Figure 4). Free-radical ROS, like hydroxyl ion radical (OH•), superoxide anion radical (O_2_^•−^), and alkoxyl (RO^•^), carbonate (CO_3_^•−^), peroxyl (RO_2_^•^), hydroperoxyl (HO_2_^•^) molecular oxygen (O_2_), and non-free radical species such as ozone (O_3_), hydrogen peroxide (H_2_O_2_), singlet oxygen (^1^O_2_), hypobromous acid (HOBr), hydroperoxy (ROOH), hypoiodous acid (HOI), hypochlorous acid (HOCl), are severely toxic to plant growth and development [73,74,75,76]. In response to heat stress, ROS are generated in different cellular parts, like the plasma membrane, mitochondria, cell wall, chloroplast, peroxisome, endoplasmic reticulum, and apoplast [77,78]. Excessive production of ROS damages molecules and compounds in plant cells like lipids, deoxyribonucleic acid (DNA), ribonucleic acid (RNA), proteins, and carbohydrates [79,80,81].

## 8. Heat Stress Causes Oxidative Stress

**Figure 5:**
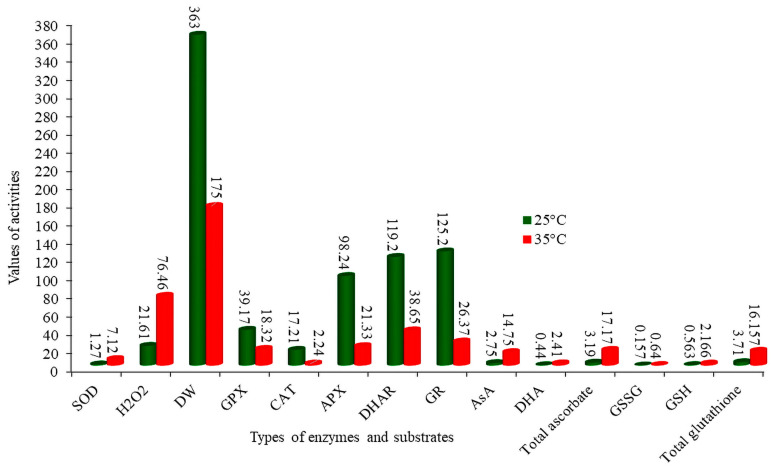
The activities of enzymes and substrates in tomato plants under heat stress. Measurement units, SOD: unit mg protein^−1^ min^−1^, H_2_O_2_: mmol g^−1^ (FW), W.D: g plant^−1^, GPX, CAT, APX, AsA, DHAR, DHA, GR, GSH, total ascorbate, and total glutathione: μmol mg-prot^−1^ min^−1^.

Tomato plants are sensitive to high temperatures, even a little beyond optimal, which causes the overproduction of reactive oxygen species (ROS) [82]. An equivalence between ROS production and antioxidants is essential for the proper growth and development of plants [83], but high temperatures are known to disrupt this equivalence in tomato plants. The tomato variety “Tmknvf_2_” was subjected to oxidative metabolism analysis at an optimal temperature of 25 °C and a high temperature of 35 °C by reference [84], focusing on superoxide dismutase (SOD), ascorbate peroxidase (APX), dehydroascorbate peroxidase (DHAR), guaiacol peroxidase (GPX), catalase (CAT), ascorbate (AsA), glutathione reductase (GR), hydrogen peroxide (H_2_O_2_), dehydroascorbate (DHA), glutathione (GSH), oxidized glutathione (GSSG), total ascorbate, total glutathione, and dry weight (DW). Generally, the activities of the CAT, APX, DHAR, GR, and GPX enzymes are enhanced in response to higher temperatures [85,86]; however, in the case of reference [84], their activities were reduced because high temperatures denatured these proteins. The concentrations of GSH, GSSG, DHA, AsA, total ascorbate, and glutathione antioxidant compounds were higher at 35 °C than at 25 °C. These substrates are utilized by CAT, APX, DHAR, GR, and GPX in the ascorbate–glutathione cycle, but their activities were reduced by higher temperatures (35 °C), resulting in a higher accumulation of these substrates (Figure 5) and increased hydrogen peroxide (H_2_O_2_) accumulation in tomato leaves. Overproduction of ROS seriously impairs plant growth, development, and yield [87]. Therefore, it is imperative to investigate oxidative metabolism in tomato plants thoroughly and develop heat-tolerant varieties.

## 9. Phenological Modifications in Response to Heat Stress

**Figure 6:**
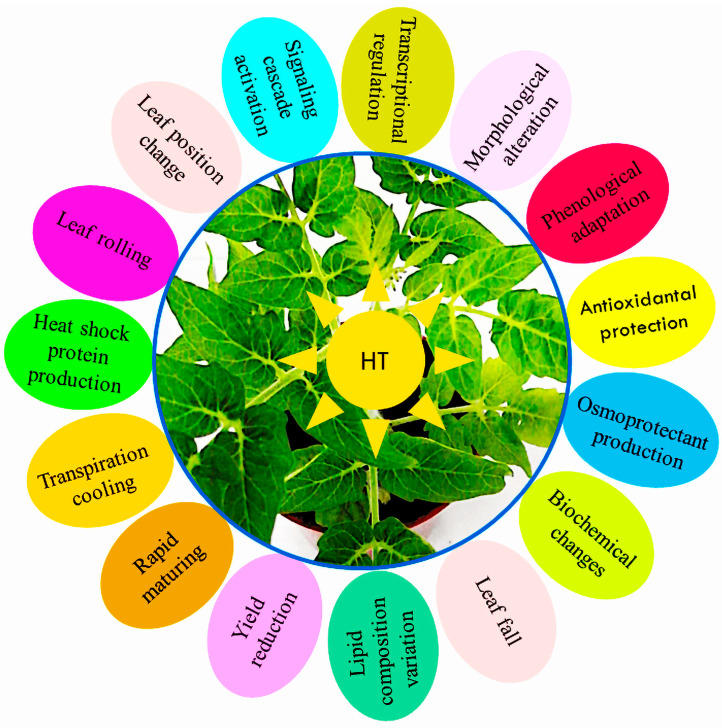
Various long- and short-term phenological changes adopted by tomato plants in response to heat stress.

Plant heat resistance refers to the ability of plants to thrive and produce the required yield under high temperatures, which is specifically linked to the plant species or potentially to the distinct organs and tissues within the same plant. Plant reactions to heat stress depend on the threshold degree, exposure period, and plant nature. The effects of heat stress on a plant’s many functioning processes, such as seed germination, development, growth, procreation, and yield, are toxic [88,89]. Under conditions of severely high temperature, serious damage to cells, even complete breakdown of cellular structures, and cell demise might occur rapidly [90]. In response to high temperatures, plants implement several short-term acclimation mechanisms and long-term evolutionary strategies for persistence (Figure 6) [91]. Among these stratagems are stomatal closure, leaf position changes, variations in the lipid configuration of the membrane, larger xylem, reduced water loss, fast maturation, increased transpiration, decreased absorption of radiation, an increase in the number of hairs on the surface, cuticle layer thickening, adoption of paraheliotropism, an increase in wax, late embryogenesis abundant proteins, transcriptional regulation, more vigorous antioxidant defense, signaling cascades stimulation, osmoprotectant, and phenological, morphological, biochemical, anatomical, molecular, and genetic adaptations [92,93]. Numerous heat-inducible genes, often referred to as heat shock genes (HSGs), exhibit upregulation in response to thermal stress. These genes encode HSPs, which are essential for plants to survive in life-threatening heat stress [94,95]. Heat shock proteins (HSPs) are biologically active only during certain plant development and growth stages, including seed germination, embryo microsporogenesis, and fruit ripening [96,97].

Under elevated temperatures, tomato plants manifest symptoms including stunted growth, aberrant development, poor photosynthesis, reduced crop output, and even plant mortality [98]. However, it is essential to note that not all genotypes of tomatoes are susceptible to high temperatures [99]. Some studies found that growing tomatoes between 21 °C and 26 °C decreased the entire carotene concentration but did not affect lycopene quantity. In contrast, cultivating tomatoes within the temperature range of 27 to 32 °C reduced ascorbate and lycopene levels while concurrently enhancing the levels of routine caffeic acid derivatives and glucosides [100]. Moreover, tomato fruit firmness and better shelf life were found in F1 hybrids, having mutant genes such as alcobaca (alc), ripening inhibitor (rin), and non-ripening (nor). These hybrids can maintain, to a greater extent, tomato hue, feel, taste, and nutritional value even when exposed to high-heat-stress conditions [101]. High temperatures during fruit development negatively affect assimilation, distribution, and shelf storage. The fruit produces several structural and functional elements throughout the ripening process, including starch and secondary metabolites that affect the interior quality of fruits [102]. The sucrose that fruit receives from the leaves as photosynthesized sugars adds to the fruit’s dry matter. A tomato’s flavor results from transforming carbs like sucrose into organic acids and aromatic compounds [103]. Environmental parameters, such as temperature, water irradiation, and photosynthesis, affect fruit quality [104]. These problems, because of heat stress, draw our attention to exploring, selecting, and utilizing cultivars of tomatoes capable of enduring high temperatures during the cultivation period. Therefore, it is critical to understand the molecular and genetic mechanisms regulating plants’ short- and long-term natural defense strategies in response to heat stress, which could be applied to regulate the heat stress problem in crops, particularly in heat-sensitive plants like tomatoes.

## 10. Heat Shock Signaling Pathway Modulation

In the face of heat stress, plants have several free and dependent pathways to perceive external and internal signals, which significantly regulate the development of responses to create resistance to cope with the situation [105]. These responses entail the overexpression of several genes and the activation of complex integrated circuits involving various pathways. Cofactors and signaling molecules such as mitogen-activated protein kinase (MAPK/MPKs), sugar compounds, and Ca-dependent protein kinases (CDPKs) play a fundamental role in activating stress-responsive genes [106,107]. However, an intrinsic study must fully elucidate and understand the signaling molecules and pathways involved in developing heat tolerance.

## 11. Heat Shock Protein (HSP) Production

Heat stress often triggers the activation of heat-inducible genes known as heat shock genes (HSGs), which produce heat shock proteins (HSPs) that are essential for a plant’s existence under very high temperatures [108]. HSPs act as chaperones to safeguard intracellular proteins from decomposition and maintain their integrity and functionality by facilitating protein folding [109]. Previously, scientists thought heat stress was the main trigger for HSP formation. However, they have since learned that many biotic and abiotic stimuli could cause HSP formation. They show up- or downregulation responses to biotic and abiotic stress situations, but further research is needed to understand signal recognition and transmission processes fully [6]. Additionally, a plant can overcome these obstacles with the help of post-transcriptional modifications, including alternative splicing and micro RNA (miRNA). Alternative splicing creates many transcripts from a single gene, while miRNA binds to mRNA to inhibit translation or induce mRNA cleavage at any location [110,111]. In plants, heat shock proteins can be divided into five categories, including small HSP20 (sHSP20), HSP60 (GroE), HSP70 (DnaK), HSP90, and (HSP100). Among these HSPs, HSP60 and HSP70 are incredibly conserved, suggesting their crucial function in the heat stress response [112]. HSP20s is a low-molecular-mass (15 to 42 kDa) family with a 90-amino acid alfa-crystallin domain (ACD) that forms a seven-stranded β-sandwich flanked by a variable N-terminal domain (NTD) with fewer to 85 amino acids and a short C-terminal extension (CTE) and is predominantly induced by heat stress in several higher plants [113].

Plants sense heat stress principally at the plasma membrane, leading to the opening of particular calcium channels, permitting calcium ions to enter the cell, and triggering the activation of mitogen-activated and calcium-dependent protein kinases, which in turn activate the heat stress response (HSR) [114,115,116]. At the time of the HSR, numerous specific genes are upregulated essentially, leading to the accumulation of a significant amount of HSPs in different cellular compartments, which play a crucial role in signaling and heat resistance mechanisms during the HSR; HSPs are commonly regulated by heat shock factors (HSFs) [117,118]. Various pathways transmit heat signals to HSFs, activating HSPs and heat-responsive genes (HRGs) and playing a significant role in plant heat adaption mechanisms, which suggests that the HSF-HSP pathway is critical in governing plant responses to heat stress [119].

## 12. Heat Shock Factor (HSF) Activation

Heat shock factors (HSFs) are activators that trigger the transcription of heat shock genes and bind to heat shock sequence elements (HSEs) found throughout the genome, which consist of a tandem array of three oppositely orientated “AGAAN” motifs or a variant of them that is less similar [120,121]. The structure of plant HSFs is very conserved and consists of several vital parts such as the oligomerization domain (OD), DNA binding domain (DBD), transcriptional activation motif (AHA), nuclear export signal (NES), and the nuclear localization signal (NLS) [122]. The oligomerization domain (OD) consists of a bipartite heptad pattern of hydrophobic amino acid residues in the HR-A and HR-B regions, and a flexible linker links it to the DNA-binding domain (DBD) [123]. The N-terminal DNA binding domain (DBD) is distinguished by a core helix–turn–helix motif that particularly attaches to the target promoter’s heat stress elements (HSEs), activating stress-inducible gene transcription [124]. The plant HSF C-terminal stimulation domain is described by short peptide motifs (AHA) that consist of giant hydrophobic and acidic amino acid residues. These residues are unique to HSFA and are absent in the HSFB and C classes [125]. The nuclear localization signal (NLS) and nuclear export signal (NES) of HSFs play a significant role in forming a nuclear entrance complex consisting of the target protein and the receptor-mediated export complex, including the NES receptor exportin-α [126]. The classification of plant HSFs into HSFA, HSFB, and HSFC is based on the number of amino acid residues inserted into the HR-A and HR-B regions and the linker length area between the DBD and HR-A and HR-B regions [127]. Class A HSFs have the transcriptional activation domain, while classes B and C HSFs lack this specific amino acid motif and cannot promote transcriptional activation alone [128,129]. It is now well-established that several HSF classes modulate HSP expression in tomato plants and have a positive regulatory role in osmotic, oxidative, thermal, anoxia, and stress tolerance, particularly by HSFA [130].

### The Heat Shock Factor A1 Class (HSFA1)

Investigations on model crop plants, including tomato [131], *A. thaliana* [132], and soybean [133], have shown that HSFA1-related genes are expressed constitutively under normal circumstances; however, their expression increases rapidly under heat stress, which designates them as significant master regulators of the heat stress response. In tomatoes, there are four members of the class HSFA1, namely, HsfA1a (Solyc08g005170), HsfA1b (Solyc03g097120), HsfA1c (Solyc08g076590), and HsfA1e (Solyc06g072750). Among these members, HSFA1a is the master regulator because of its consistent expression in control and heat stress (HS) conditions across all tissues. On the other hand, HSFA1c and HSFA1e are typically significantly expressed in red ripe fruits, while HSFA1b is strongly stimulated in all fruit stages [134]. HSFA2 plays a crucial role in the priming mechanism of tomato plants, which is responsible for maintaining pollen thermotolerance throughout the process of microsporogenesis [135]. A previous study provided evidence that the expression reduction in HSFA2 resulted in a decrease in the viability and germination rate of pollen exposed to HS during the meiosis and microsporogenesis phases, which supports the notion that it plays a crucial role in maintaining thermotolerance [136]. The expression levels of tomato HSF genes, namely, SlyHSF01, SlyHSF8, SlyHSF9, SlyHSF10, and SlyHSF11, have been observed to be significantly higher in leaf tissues under a high temperature (45 °C) compared with a control (30 °C) situation [137]. The cytoplasm is the site for tomato HSFA3 (Solyc09g009100) expression under a controlled environment, while the nucleus is the site of its expression under HS circumstances [138]. According to reports, tomato HsfA4s (Solyc07g055710, Solyc03g006000, and Solyc02g072000) significantly boost the expression of HS genes, while HSFA5 (Solyc12g098520) is a particular inhibitor of HSFA4 action [139,140]. A reduction in HSFB4a ((Solyc04g078770) expression and a boost in HSFA7 levels regulate thermo-tolerance in tolerant tomato cultivars [141]. The overexpression of SUMO E3 ligase (SlSIZ1) in tomatoes led to an enhanced heat tolerance by regulating the activities of HSFA1 and promoting the accumulation of HSP70 [142]. In a heat-resistant tomato cultivar (CLN1621L), the gene notabilis (Solyc07g056570) and acyl-sugar acyltransferase (Solyc09g014280) exhibit upregulation as positive regulators of HS tolerance, while the gene Pin-II proteinase inhibitor (Solyc03g020030) shows downregulation as a negative regulator of thermotolerance, indicating that the inverse expression of these genes encodes enzymes and proteins that play significant roles in mitigating heat stress [143].

## 13. Involvement of Omics Approaches 

Omics technologies are distinguished by their systematic investigation and analysis of extensive datasets that capture the entirety of a biological system’s structure and function at a specific level, which has significantly transformed the approaches used to study biological systems [144]. Multi-omics strategies involve techniques such as transcriptomics, genomics, metabolomics, proteomics, epigenomics, proteogenomics, lipidomics, interactomics, ionomics, phenomics, and bioinformatics, which produce a significant amount of data that can be utilized to understand the physiological and molecular mechanisms functioning in plants under stresses and devise effective strategies for mitigating the adverse impacts of such stresses [145,146]. However, relying exclusively on a single omics approach is inadequate to fully elucidate the complexities of plant responses to abiotic stresses, particularly HS. The utilization and incorporation of multi-omics methodologies are necessary to achieve promising results. Hence, integrating multi-omics methods is essential for satisfactory inferences [147,148].

### 13.1. Genomics

Genomics research explores a genome’s structure, function, evolution, mapping, and changes. At the same time, the latest advances in molecular biology have quickened the rate of high-throughput genome sequencing, genomic characterization, and gene expression analysis [149]. Functional genomics involves the analysis of partial or unbiased genome sequencing data to elucidate gene functions and interactions, which is achieved through a forward approach consisting of investigating randomly obtained mutants of a particular phenotype and identifying the responsible gene or a reverse approach by disrupting a known gene to examine the organism’s phenotype [150,151]. Genome-wide association studies (GWASs) involve the comprehensive analysis of a complete genome to uncover DNA changes associated with a particular trait [38]. The main objective of GWASs is to determine genomic regions related to agronomic or morphological characteristics and any phenotypes that may serve as markers, genes, or QTLs for gene identification, introgressive hybridization, and marker-assisted breeding (MAB) [152,153]. GWASs revealed the upregulation of SlTFT6, a gene belonging to the Sl14-3-3 family, which improved thermotolerance in tomato plants [154]. Structural genomics focuses on elucidating the three-dimensional configuration of genes to ascertain their identity, position, and arrangement along the chromosome [155]. Genomic selection represents an innovative approach to enhancing quantitative traits by leveraging marker and phenotypic data obtained from observed populations, thereby evaluating the influence of all genetic loci [156]. Genome sequencing and mapping comprise several systems, such as the Roche 454GS FLX Titanium or Illumina Solexa Genome Analyzer, which are considered next-generation sequencing (NGS) platforms and have significantly reduced the cost and time required for sequencing compared with traditional methods like the Sanger method [157]. These platforms have provided comprehensive information regarding the characteristics of genomes, including coding and non-coding genes, GC contents, repetitive elements, and regulatory sequences, which have facilitated the development of improved crop varieties such as tomato, rice, wheat, maize, sorghum, and soybean [158,159]. Molecular markers, also known as genetic markers, are segments of DNA that may detect changes in a population’s DNA or polymorphisms, including deletions, insertions, and substitutions of bases [160]. Various molecular markers, such as random amplified polymorphic DNA (RAPD), simple sequence repeats (SSRs), sequence-tagged sites (STSs), restriction fragment length polymorphism (RFLP), single-nucleotide polymorphism (SNP), and amplified fragment length polymorphism (AFLP), have been recently identified as valuable tools for identifying polymorphisms in plants [161]. The investigation of comparative genomics involves the alignment of biological sequences and the identification of conserved sequences, which reveals significant synteny among related species [162] and enables the detection of small-scale changes within different genomes, including protein-coding regions and their impact on protein structure and function [163].

### 13.2. Transcriptomics

The term “transcriptome” covers the complete collection of ribonucleic acid (RNA) molecules within an organism or a particular cell type, which mainly ranges from protein-coding messenger RNA (mRNA) to various non-coding RNAs such as transfer RNA (tRNA), long non-coding RNA (lncRNA), ribosomal RNA (rRNA), primary microRNA (pri-miRNA), and small nuclear RNA (snRNA) [164,165,166]. The transcriptomic approach covers multiple facets of RNA-seq evaluation, especially experimental design, quality control, read alignment, quantification of gene and transcript levels, visualization, differential gene expression, alternative splicing, functional analysis, gene fusion detection, and expression quantitative trait loci (eQTL) mapping [167,168]. The primary focus of transcriptomic research is to examine gene transcripts or RNA linked to a plant’s phenotypic expression under various stress conditions [169] by employing a range of techniques, including serial analysis of gene expression (SAGE), DNA microarrays, and high-throughput technologies based on next-generation sequencing (NGS) for conducting digital gene expression (DGE) and RNA sequencing (RNAseq) [170,171]. The transcriptomic analysis of microspores from a heat-tolerant tomato cultivar (cv. Hazera 3042) revealed elevated levels of heat-responsive gene expression, specifically LeHSFA2, LeHSP17.4-CII, homologs of LeHSP90 (*Laternula elliptica*), and AtVAMP725 (*A. thaliana*), compared with a control [172]. The transcriptomic study findings indicated a notable increase in the expression of SAUR (small auxin upregulated RNA) family proteins, MYB (myeloblastosis viral oncogene homolog) transcription factors, and NAC (no apical meristem) domain proteins in response to arid environmental conditions. Furthermore, it was observed that the heat-tolerant line exhibited a significant inclusion of heat shock proteins and proteinase inhibitors [173]. The transcriptomic analysis of tomato plants subjected to heat stress at temperatures of 35/25 °C, in conjunction with specific nitrogen fertilizer levels, showed a significant upregulation of genes, including cell wall invertase (CWINV2; Solyc10g085650.2, Solyc10g085640.1) and sucrose transporter (SUT1; Solyc11g017010.2), while hexokinase 2 (HK2) (Solyc06g066440.3), SWEET2 (Solyc07g062120.4), and SWEET1 (Solyc04g064610.3) exhibited downregulation [174].

### 13.3. Metabolomics

Metabolomics is the scientific investigation of naturally occurring tiny, low-molecular-weight metabolites, including carbohydrates, fatty acids, amino acids, steroids, and lipids, which play distinctive roles in interpreting cellular biochemistry [175,176]. The function of a metabolite can be significantly altered by minor alterations in its chemical structure and the presence of external abiotic or biotic stimuli [177]. Metabolomics inquiry offers distinct advantages over other omics because metabolites are the downstream products of gene and protein activities, which determine the impact on biological phenotype and other physiologic processes [178]. Plant metabolites can be primary metabolites, which are crucial for growth and significantly impact physiological processes, and secondary metabolites, which are vital for defense mechanisms in response to various stressors [179,180]. A variety of advanced techniques exist for the analysis of plant metabolites, including gas chromatography (GC), high-performance liquid chromatography (HPLC), thin-layer chromatography (TLC), paper chromatography (PC), nuclear magnetic resonance (NMR), metabolic flux analysis (MFA), extracellular flux analysis (EFA), direct-inject mass spectrophotometry (DIMS), Fourier transform infrared spectroscopy (FTIR), capillary electrophoresis (CE), and mass spectrometry (MS), which have proven to be valuable tools for researchers [181,182]. A metabolic investigation of tomatoes under elevated temperatures and relative air humidity revealed the disruption of enzymes involved in sucrose metabolism, resulting in a decrease in the fruit-soluble sugar content. Conversely, an increase in the activities of enzymes associated with phosphopyruvate carboxylase (PEPC), mitochondria aconitase (MDH), and citrate synthetase (CS) led to an elevated content of malic acid [183]. Metatomic analysis has shown a significant association among sucrose, glucose, fructose, the TCA cycle, starch production, and HS tolerance [184]. Liquid chromatography–mass spectrometry (LC-MS) identified an increased accumulation of secondary metabolites, specifically flavonoids, within the pollen microspore of tomatoes under heat stress [185]. A metabolic analysis of tomatoes using gas chromatography–mass spectrometry (GC-MS) revealed that heat treatment mitigated the effects of chilling on fruits by modifying the concentrations of several fruit metabolites, including arabinose, fructose-6-phosphate, valine, and shikimic acid, in the chilled samples as compared with a control [186].

### 13.4. Proteomics

Proteomics comprehensively explores protein composition, structure, expression, modification status, connections, and interactions among proteins [187]. Basic proteomics techniques include one-dimensional (1D) and two-dimensional (2D) gel electrophoresis (2-DE) methodologies [188]. Several other high-throughput screening technologies such as shotgun proteomics (SP), nanoflow liquid chromatography coupled to tandem mass spectrometry (nLC-MS/MS) [189], stable isotope labeling by amino acids in cell culture (SILAC) [190,191], multidimensional protein identification technology (MudPIT) [192], isobaric tags utilized in relative and absolute quantitation (ITRAQ) [193,194], the Western blot (WB) technique [195], multiple reactions monitoring mass spectrometry (MRM-MS) [196], and tandem mass tags (TMTs) [197,198] are available for utilization according to research objectives. Proteomic analysis of the tomato revealed better pollen tolerance to heat stress following ethephon pre-treatment by increasing protein abundance in processes of protein synthesis, degradation, the tricarboxylic acid cycle, and RNA regulation [199]. Another proteome analysis of tomatoes subjected to high-light-induced stress revealed a notable presence of oxygen-evolving complex and PSII complex proteins, including PsbH, PsbS, PsbR, and Psb28, within the leaf zone that exhibited the maximum damage [200]. Tandem mass tag (TMT)-based analysis of pollen mother cells at the initial anther developmental stage in the Maxifort tomato variety revealed the upregulation of 96 proteins including heat shock proteins, calreticulin, and exocytosis associated with protein folding/refolding/targeting/removal along with the secretion of aggregated and damaged proteins/peptides and the downregulation of 158 proteins active in ubiquitin-mediated protein breakdown, antioxidant mechanisms, and the metabolism of lipids and carbohydrates [201].

## 14. Genome Editing Strategy Application

Genome editing has emerged as a promising tool in tomato breeding, offering the potential for immense success and fully utilizing genome information and phenotyping technologies. It is divided into two major approaches, first, as site-directed nuclease (SDNs) and, second, as oligonucleotide-directed mutagenesis (ODMs) involved in creating mutations in the genome [202]. Its application could enhance HS resistance by introducing mutations into negative regulatory genes, which have a pivotal role in tomato HS tolerance [203]. It entails the utilization of several DNA-cleaving enzymes, known as nucleases, specifically designed to cleave the DNA at a pre-established site through diverse DNA binding systems. Several techniques can be applied to carry out specific DNA cleavages, such as zinc finger nucleases (ZFNs), mega-nucleases (MNs), clustered regularly interspaced short palindromic repeat (CRISPR)-associated proteins (CRISPR/Cas), and transcription activator-like effector nucleases (TALENs). These are entitled site-directed nucleases (SDNs), representing the fundamental concept of using a DNA-cutting enzyme (nuclease) to create a specific DNA break at a particular location [204,205]. CRISPR/Cas systems are further divided into classes 1 and 2 based on effector molecules. The class 1 system has multiple effector molecules and is subdivided into three types, including I, III, IV, and 12 subtypes found in 90% of the CRISPR loci in bacteria and archaea targeting DNA and RNA. The class 2 system is characterized by a single effector molecule, with three types, including II, V, and VI, and nine subtypes, which represent 10% of the CRISPR loci targeting DNA and RNA and are found in bacteria. The most prevalent CRISPR/Cas systems utilized for gene editing are type II-A Cas9 from Streptococcus pyogenes and type V-A Cas12a (Cpf1) from Acidaminococcus sp. and Lachnospiraceae [206,207,208]. The CRISPR/Cas9-mediated removal of SlUDPGT52 resulted in improved drought tolerance because of increased reactive oxygen species (ROS) scavenging [209]. The efficacy of CRISPR/Cas9 in facilitating the introduction of de novo domestication of elite features from wild relatives to the cultivated tomato, as well as the reverse process, has been demonstrated. CRISPR/Cas9 technology has been utilized to manipulate and examine a range of attributes about tomatoes, including leaf, stem, and male sterility, parthenocarpy, fruit maturation, quality, nutrition, heat, drought, salinity stress, carbon–nitrogen metabolism, and herbicide resistance [210,211]. Genome editing technology has contributed to the heat-tolerant breeding of tomatoes via the identification of critical genes associated with acquired thermotolerance mechanisms, including SlIAA9, HsfA2, JA/COI1, HsfB1, and SlAGL6 [212]. It has improved tomato resistance by regulating genes such as lateral organ boundaries domain (SlLBD40) (Solyc02g085910), mitogen-activated protein kinase (SlMAPK3), and cytidine base editor (CBE) against biotic and abiotic stressors [213].

## 15. Development of Heat-Tolerant Tomato Varieties

There has been a global demand for the development of heat-tolerant varieties to effectively respond to both present and anticipated rises in heat stress. However, breeding for heat tolerance has encountered challenges related to the intricate nature of heat stress and plant reactions and the limited comprehension of the genetic underpinnings of heat tolerance characteristics [214]. The efficacy of heat-tolerant breeding is contingent on the proficient determination and description of constituent qualities that underlie heat resistance processes in the presence of heat stress, as well as the comprehensive understanding of their genetic structure throughout both the vegetative and reproductive phases [215,216]. Affordable and technologically sophisticated high-throughput genotyping is being applied; however, accurate phenotyping is a significant barrier to understanding the genetic basis of required but intricate traits, which slows down breeding programs [217,218]. Effective plant breeding programs have to prioritize the development of phenotyping techniques that are cost-effective, precise, reliable, less labor-intensive, reproducible, and easily applicable, targeting traits such as increased yield, resistance to biotic and abiotic stress, improved quality, photosensitivity, synchronous maturity, and detoxification ability. To develop tomato genotypes resistant to high temperatures, it is crucial to examine cultivated and wild tomato genetic resources thoroughly. To create heat-resistant tomato varieties that have high production and yield, it is essential to comprehend the genetic architecture of heat-tolerance traits to effectively regulate the increase in metabolites, osmoprotectant, photosynthetic activity efficiency, membrane stability, the number of flowers per inflorescence, inflorescence number, pollen number, female fertility, pollen viability, fruit set, fruit number, and fruit weight and the decrease in canopy temperature, style protrusion, and style length [219,220,221]. Previous breeding projects have not derived significant benefits from the sizeable range of wild tomatoes, mainly because of problems such as progeny sterility, self-incompatibility, and linkage drag [222]. For breaking linkage drag, various techniques, including chromosome segment substitution lines (CSSLs), advanced backcross quantitative trait loci (QTL) analysis, and backcross inbred lines (BILs), could be applied to generate lines that possess small fragments of donor parent chromosomes [223,224,225]. A practical approach in tomato breeding efforts to enhance resistance to abiotic and biotic stressors is incorporating native germplasm and wild relatives into existing varieties by introducing novel allelic combinations. Multiple tomato introgression lines have been developed by using wild cousins such as *Solanum pimpinellifolium*, *Solanum habrochaites*, and *Solanum pennellii*, which exhibit resistance to abiotic and biotic stressors [226,227,228].

## 16. Genetic Resource Development

Genetic resources or germplasm includes plants, parts of plants, and seeds, which are significant for breeding, research, and conservation. For example, seeds of an ancient heirloom tomato variety passed down to current time are just seeds produced by a gardener or company, but they are germplasm when part of a breeding program for new variety development, collected for the preservation of the genetic diversity, or preserved as genetically governed traits [229]. Lycopersicum tomato species are diploid (2n = 2x = 24) with similar chromosome numbers and structures [230] that produce perfect hermaphrodite flowers and have a complete range of mating systems from autogamous *L. cheesmanii* and *L*. *parviflorum* to obligately outcrossed self-incompatible biotypes of *L. chilense*, *L. hirsutum*, *L. peruvianum*, and *L. pennellii* [231], while the tendency of self-fertility with different levels of facultative outcrossing is present in *L. chmielewskii*, *L. esculentum*, *L. pimpinellifolium*, and the self-compatible biotypes of *L. hirsutum* and *L. pennellii* [232]. In the quest for specific genetic traits, contemporary and prospective researchers and crop breeders must have full access to landraces, diverse varieties, and relevant wild species. Multiple institutes have developed tomato genetic resources to cater to the needs of researchers and breeders studying heat tolerance and other agronomic features. About 62.8 thousand tomato accessions, both wild and domesticated varieties (*L. esculentum*), are present in gene banks across the globe and are ready to be used for genealogical research [233]. The Tomato Genetics Resource Center (TGRC) at the University of California (Davis) (http://tgrc.ucdavis.edu/, accessed on 11 December 2023), the United States of America, is a well-recognized and valuable repository of various germplasm and wild species. The World Vegetable Center (http://seed.worldveg.org, accessed on 11 December 2023), located in Taiwan, China, maintains a vast assortment of around 8835 tomato accessions, of which 6676 are readily available for procurement upon request. A wide variety of tomato genetic resources have been collected by the National Agriculture and Food Research Organization (NARO) gene bank (https://www.gene.affrc.go.jp, accessed on 22 January 2024) and the National Bio-Resource Project (NBRP) of tomato (https://tomato.nbrp.jp, accessed on 24 January 2024) in Japan. The National Bio-Resource Project (NBRP) maintains a collection of more than 10,000 Micro-Tom mutants that have been generated using the techniques of gamma-ray irradiation and ethyl methane sulfonate (EMS) mutagenesis [234,235]. The Micro-Tom plant is a model for investigating fruit production and its ability to withstand different abiotic and biotic challenges [236]. Data about Micro-Tom mutants can be retrieved from the TOMATOMA database, found at http://tomatoma.nbrp.jp/index.jsp (accessed on 8 February 2024) [237]. Several other genetic resources are available from which seeds or genetic material could be obtained, including the Solanaceae Genomics Network (SGN, http://solgenomics.net/, accessed on 8 February 2024), the United States Department of Agriculture (USDA) (https://www.usda.gov/, accessed on 14 February 2024), the Tomato Genetics Cooperative (TGC) (https://tgc.ifas.ufl.edu/, accessed on 17 February 2024) at the University of Florida, USA, the Ohio State Tomato Breeding and Genetics Program (OSTBGP) (https://tomato.cfaes.ohio-state.edu/, accessed on 17 February 2024), Vavilov Institute, Russia (VIR) (https://www.vir.nw.ru/en/, accessed on 3 March 2024), and Instituto de Investigaciones Fundamentales en Agricultura Tropical (INIFAT), Cuba [238,239]. These genetic resource reservoirs could be accessed and explored to obtain genes for tomato heat resistance improvement and other targeted breeding programs.

## 17. Conclusions and Future Aspects

The average global temperature has significantly increased because of global climate change, which has also put food security and agricultural output at risk [240]. Reduced photosynthesis, decelerated growth and development, and reduced nutrient uptake are just a few of the physiological and biochemical processes upset by heat stress in tomatoes, resulting in yield losses [220,241]. Over the next several years, it is anticipated that the damaging consequences of heat stress will get worse. Uncertainty surrounds the magnitude of the potential consequences associated with global warming. Changes can have both direct and indirect impacts on food production conditions. Direct changes can lead to significant changes in food production, resulting in increased mortality rates because of floods, storms, heat waves, and droughts. On the other hand, indirect effects may include unemployment in rural areas requiring specific climate conditions for crop growth, such as cultivating tomatoes in open fields [242,243].

Environmental change, particularly a rise in ambient temperatures, substantially affects plant growth, development, production, and yield, leading to a severe decline in crop yield and jeopardizing international food security. Increasing heat stress disrupts various physiological and biochemical systems in tomato plants. Tomato seed pollen viability and root development are significantly affected by elevated temperatures in different parts of the world. The emerging data indicate that reactive oxygen species (ROS) cause cellular oxidative damage but also serve as signaling molecules in the heat stress response (HSR), triggering adaptive responses. However, the exact mechanism underlying the interconnections among various signaling pathways linked with ROS has not yet been fully understood. Understanding the interaction between ROS and redox signals and identifying the precise redox pathways activated in different cell compartments is essential for adjusting HSR in response to varying HS intensity and duration levels. The molecular processes behind the pollen heat-stress response and thermotolerance remain largely unexplored. In the context of escalating global warming, there is an urgent need for molecular and genetic research to ascertain the genes responsible for conferring heat tolerance in tomatoes, thereby mitigating the detrimental effects of high temperatures. The primary objective of high-throughput phenotyping should be to investigate several aspects of plant physiology, including canopy temperature, pollen viability, photosynthetic efficiency, membrane thermostability, sugar content, and osmoprotectant activity, to obtain full inside knowledge.

In our opinion, to enhance the overall resilience of tomatoes against heat stress, it is crucial to elucidate the molecular and physiological mechanisms underlying the negative correlations among seed germination, plant growth, development, pollen viability, fruit sets, fruit size, fruit weight, other agronomic traits, and thermal stress. Collecting diverse tomato genetic resources, including various cultivars and wild species, would be valuable for future genetic engineering, particularly in developing heat-resistant tomato plants. Including wild tomato species in breeding programs incurs some drawbacks since introducing genes from wild relatives into advanced lines might alter the already established horticultural features owing to linkage drag. Transgenic technology has the potential to serve as an advantageous instrument for enhancing the heat stress resistance of tomatoes, especially when integrated with conventional techniques. Integrating marker-assisted breeding with high throughput phenotyping can significantly improve the breeding performance of tomatoes in terms of heat resilience. Understanding the genetic foundations of novel populations is of utmost importance, including approaches like chromosomal segment substitution lines (CSSLs), introgression lines (ILs), backcross inbred lines (BILs), and mutants for trait identification. Genome editing could identify the molecular mechanism of heat stress transcription factors and enhance heat tolerance features, like increasing the number of inflorescences and flowers per inflorescence.

Despite certain advancements in translational genomics, particularly with the backing of the gene-editing technology CRISPR/Cas9, some significant difficulties remain, for example, several features subject to quantitative regulation require several genes. Hence, it is imperative to manipulate several new genes to induce new desired phenotypes in modified tomato crops. Further challenges include the lack of effective delivery routes for gene editing reagents such as mRNA (sgRNA), DNA plasmid, and ribonucleoprotein (RNP), technical bottlenecks, and ethical concerns. Moreover, there is a lack of comprehensive genetic data regarding the necessary dietary components, and generating accurate alterations in DNA sequences is challenging. Nevertheless, several gene-editing techniques offer effective and precise gene editing of plants, including base editors, replicons, and targeted non-homologous insertions. The continuous progress in sequencing technology can be utilized to find reference genome sequences for previously unknown tomato wild cousins, which will serve as a great approach to exploit the genetic variability in these species. Genome editing facilitates the development of novel domestication tactics that selectively utilize tomato relatives. Establishing more vibrant collaboration between private plant breeding enterprises and public sector gene banks at regional, national, and worldwide levels is essential. It has significant benefits, particularly in enhancing the conservation and utilization of tomato genetic resources.

**Figure 7:**
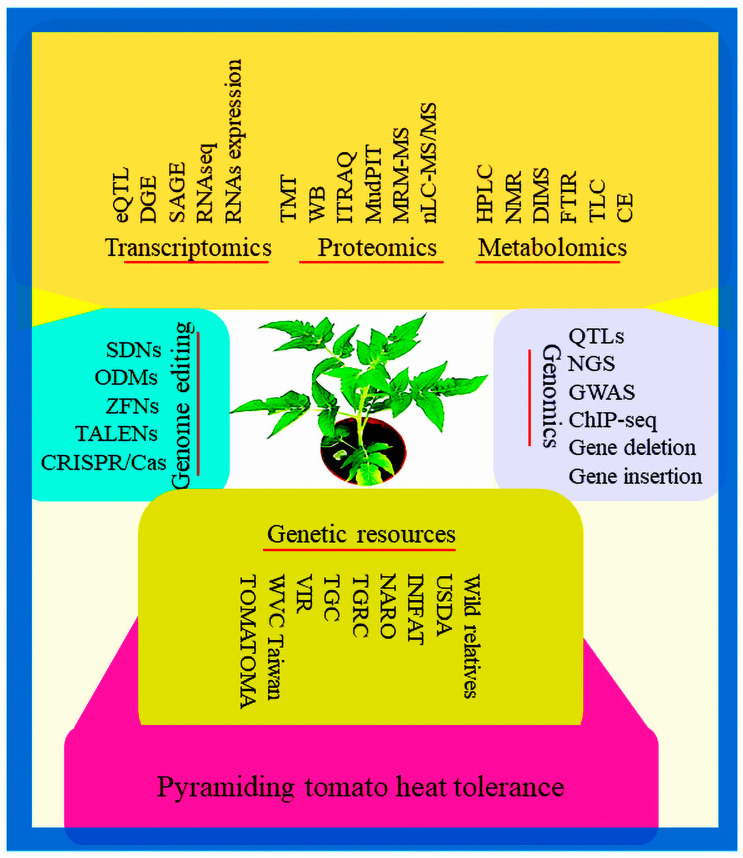
Improving heat tolerance in tomatoes through integrated approaches, including genomics, transcriptomics, proteomics, metabolomics, gene editing, and genetic resources.

A holistic approach is required to comprehensively elucidate the causes of tomato susceptibility to heat stress and the development of heat-resistant varieties in the interfaces of continuously increasing global temperature. So, integrated strategies (Figure 7) based on sophisticated technologies involving high-throughput genotyping, genome editing, and multi-omics approaches like transcriptomics, genomics, metabolomics, proteomics, epigenomics, proteogenomics, lipidomics, interactomics, ionomics, phenomics, bioinformatics genetic engineering, genetic resources collection, preservation, and utilization would enable researchers and breeders to develop heat-tolerant tomato varieties with capabilities to combat increasing temperature stress for a long time.

## Data Availability

All the necessary data are included in this manuscript.
